# Innate Immune Regulation Under Magnetic Fields With Possible Mechanisms and Therapeutic Applications

**DOI:** 10.3389/fimmu.2020.582772

**Published:** 2020-10-22

**Authors:** Hong Lei, Yi Pan, Rongqian Wu, Yi Lv

**Affiliations:** ^1^ National Local Joint Engineering Research Center for Precision Surgery and Regenerative Medicine, The First Affiliated Hospital of Xi’an Jiaotong University, Xi’an, China; ^2^ Center for Spintronics and Quantum Systems, State Key Laboratory for Mechanical Behavior of Materials, Xi’an Jiaotong University, Xi’an, China; ^3^ Department of Hepatobiliary Surgery, The First Affiliated Hospital of Xi’an Jiaotong University, Xi’an, China

**Keywords:** immune regulation, magnetic fields, macrophages polarization, iron metabolism, paramagnetic nanoparticles

## Abstract

With the wide applications of magnetic fields (MFs) in medicine, researchers from different disciplines have gained interest in understanding the effect of various types of MFs on living cells and organisms. In this paper, we mainly focus on the immunological and physical aspects of the immune responses and their mechanisms under different types of MFs. Immune cells were slightly affected by low-frequency alternating MFs but were strongly influenced by moderate-intensity MFs and high-gradient MFs (HGMFs). Larger immune cells, such as macrophages, were more sensitive to HGMFs, which biased the cell polarization into the anti-inflammatory M2 phenotype. Subject to the gradient forces of varying directions and strength, the elongated M2 macrophage also remodeled the cytoskeleton with actin polymerization and changed the membrane receptors and ion channel gating. These alterations were very similar to changes caused by the small GTPase RhoA interference in macrophage. Regulation of iron metabolism may also contribute to the MF effects in macrophages. High MFs were found to regulate the iron content in monocyte-/macrophage-derived osteoclasts by affecting the expression of iron-regulation genes. On the other hand, paramagnetic nanoparticles (NPs) combined with external MFs play an important role in T-cell immunity. Paramagnetic NP-coated T-cells can cluster their T-cell receptors (TCRs) by using an external MF, thus increasing the cell–cell contact and communication followed by enhanced tumor killing capacity. The external MF can also guide the adoptively transferred magnetic NP-coated T-cells to their target sites *in vivo*, thus dramatically increasing the efficiency of cell therapy. Additionally, iron oxide NPs for ferroptosis-based cancer therapy and other MF-related therapeutic applications with obstacles were also addressed. Furthermore, for a profound understanding of the effect of MFs on immune cells, multidisciplinary research involving both experimental research and theoretical modeling is essential.

## Introduction

The magnetic field (MF) is an elementary factor for the survival of any organism; e.g., the geomagnetic field that has an intensity of around 50 μT serves as a natural stimulation for many physiological processes in a living organism. In recent decades, MF-related techniques have found wide applications in medicine, such as magnetic resonance imaging (MRI). New MRI with the MF strength of 10.5 T has been used in preclinical trials (1). MF-based surgical techniques are also getting popular, such as magnetic compression, navigation, anchor, levitation, and tracer techniques in both animal experiments and clinical surgeries (2–7). With the wide applications of MFs in medicine, researchers from different research fields, such as biology, immunology, medicine, physics, chemistry, etc., have gained great interest in understanding the effect of various types of MFs on living cells and organisms. The biological effects of static magnetic fields (SMFs) are reviewed by Zhang et al. (8). However, such effects on living cells are quite different depending on parameters of the MF, such as homogeneity, intensity, and exposure time.

**Figure 1:**
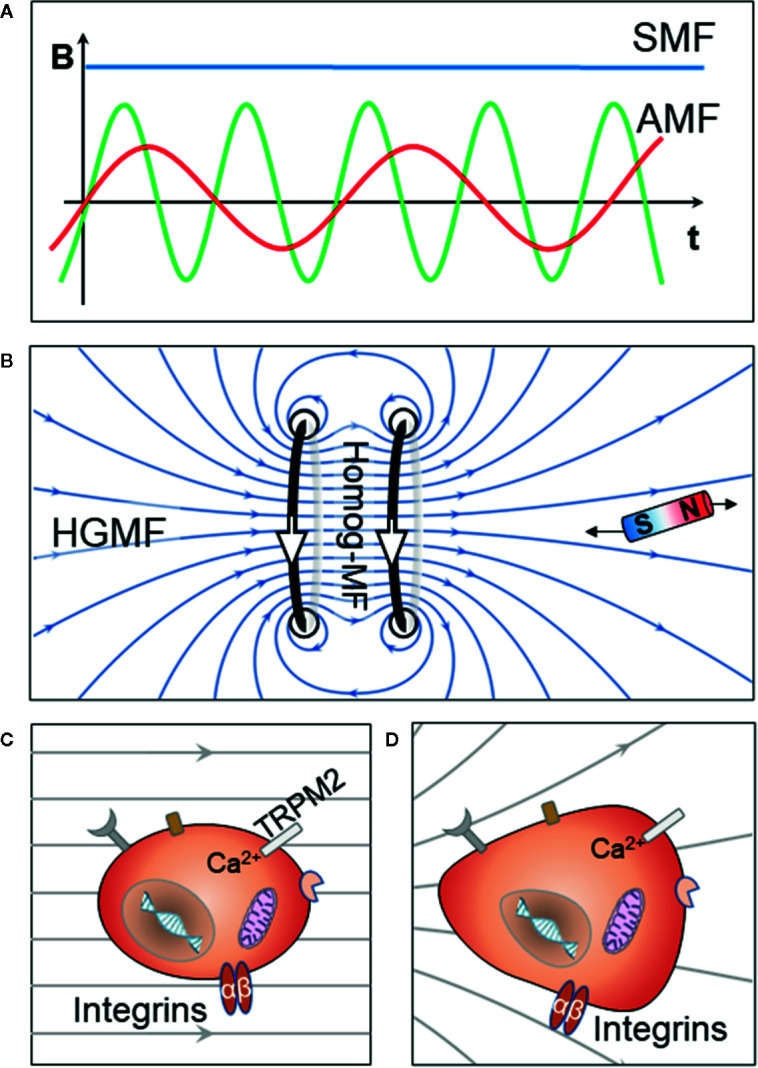
Schematic representation of the types of MFs and their effect on living cells. **(A)** An SMF is created by a magnet or a coil with steady current, and the intensity of an AMF is time-varying. “t” represents time, and “B” represents the intensity of the MF. **(B)** The high MF created by a Helmholtz coil (dark) is shown. The homogeneous MF region is located in the center, and HGMF is located in the off-center region at both sides. The small bar magnet receives a gradient net force with a stronger force at the side near Helmholtz coil. **(C)** The cell components, such as mitochondria, chromatin, and DNA, could be affected by strong homogenous MFs. **(D)** Living cells, especially with large size, get stronger mechanical forces in HGMFs. Within the macrophage, different cell components are subject to gradient forces of varying directions and strength; thus, the cells would reshape to adapt to the force and eventually end up balanced but distorted.

Immune regulation plays an important role in almost every physiological process. In recent years, more and more studies show that MFs have some effects on the immune system. Wyszkowska et al. report that low-frequency MF exposure could significantly increase the plasma inflammatory parameters in rats, and other types of MFs contribute to the anti-inflammatory and tissue repair processes (9, 10). The effect of MFs on immune responses and regulation has already gained more attention from various scientific communities. The SMF is normally created by a magnet or a coil with a steady current. In contrast, an alternating magnetic field (AMF) is time-varying and is generated when an alternating current passes through a coil, the MF intensity or strength is alternating at certain frequency (**Figures 1A, B**). Depending on the space distribution of its intensity, the SMF could be homogeneous SMF or high-gradient MF (HGMF). In general, low-frequency AMF is reported to promote the activation of innate immune cells with inflammatory responses; on the other hand, moderate frequency SMF, including gradient SMF, is shown to promote anti-inflammatory responses (11, 12). Thus, MF-related therapeutic approaches for inflammatory diseases and cancer have been further investigated in the laboratory and some even in the clinics (13–15). In this paper, we focus on immunological and physical aspects of the different immune responses and their mechanisms under different types of MFs. MF-related immune therapy, together with the challenges in clinical translation, are also addressed.

## Immune Reactions Under Various Types of MFs

The immune system consists of two distinct but intimately correlated systems: the innate and adaptive immune systems. The two systems cooperate intensively to provide a homeostatic “protective” environment against infections and injuries. We summarize the reactions of both the innate and the adaptive immune cells to different types of MFs in **Table 1**. Generally, upon exposure to a very low-frequency AMF (50–75 Hz, 0.8–7 mT), the innate immune cells get more activated and initiate the inflammatory responses in mice and rats (9, 16–21). Natural killer cells (NK cells) are found to be more cytotoxic under 0.4 T SMF (28); myeloid-derived suppressor cells (MDSCs) are repolarized under the influence of magnetic nanoparticles (NPs) from an immunosuppressive phenotype to a pro-inflammatory phenotype for glioma treatment (27). The effects of superparamagnetic iron oxide MFs accelerate dendritic cell (DC) maturation with an increased expression of MHC-II, CD80, and CD86 (24). Exposure of granulocytes from healthy human blood samples to gradient SMF resulted in the production of reactive oxygen species (ROS), which is found to be associated with the exposure time and orientation of the MF (26). In the following paragraph we summarize the effects of MFs on monocytes and macrophages, which are the most studied innate immune cells under MF exposure. The adaptive immune cells are not extensively studied as they are relatively less affected by MF exposure compared to the innate immune cells. The effect of MFs on T-lymphocytes is mainly observed during cell activation under extra stimuli (29–31, 34). Interestingly, an external MF can significantly induce T-cell receptor (TCR) clustering of the T-cells coated with iron-dextran NPs, thus leading to TCR/CD3 aggregation; the strong and sufficient signal thereby enhances the downstream events for T-cell expansion. Such strategy drove 10-fold T-cell expansion after one week (32). To date, no difference in B-cell differentiation and antibody production has been reported under MF treatment (33).

**Table 1:** Effects of MFs on immune cells.

Immune cells		MFs	Effects
Innate Immune Cells	Monocytes	6mT SMF	Mitochondria localized near nucleus; intracellular Ca^2+^ ↑ (16);
	Macrophages	LF-MF (50 Hz, 7 mT; 60Hz, 0.8mT; 50Hz, 1.0mT; 75Hz, 1mT)	IL-1β, IL-2, IL-6, TNF-α, NO, ROS↑ (in rats, RAW264.7 MΦ, bone marrow (BM) derived-MΦ) (9, 17–19),
		Constant MF (60 μT) + alternating MF (100 nT)	TNF-α, IFN-γ ↑ in mice (20)
		Space MF (around 0.5μT)	Iron in RAW264.7 MΦ↑ ROS↑ (21)
		Superparamagnetic scaffold in SMF	MΦ in superparamagnetic scaffolds → M2 Φ (IL-4, IL-10) (22)
		0.6T SMF	M2 polarization, wound closure, re-epithelialization, revascularization ↑ in diabetic mice (10);
		HGMF (10^8^-10^9^ m^−1^)	M2 polarization (anti-inflammatory effects); cytoskeletal rearrangements (11, 23),;
	DCs	Superparamagnetic iron oxide + MF	BM-DC maturation↑; MHC II, CD80 and CD86 expression↑ (24)
	Granulocytes	electromagnetic radiation (4 - 4.34 GHz, 16 min, human blood samples)	Number of viable neutrophils ↓ levels of IL1β and TNFα↑ (25)
		Gradient SMF (max: 60 mT, exposure time 15, 30, 45 min, human blood samples)	ROS metabolic oscillations affected, depending on the exposure time and orientations of MF (26)
	MDSCs	magnetic nanoparticles	Repolarization from immunosuppressive phenotype to a pro-inflammatory phenotype for glioma treatment (27)
	NK cells	0.4T SMF	Improve the killing activity of the NK cells (28)
Adaptive Immune Cells	T-cells	SMF+ power frequency MF	23.4μT SMF no influence on Ca^2+^; 16Hz/42.1μT power frequency MF + 23.4μT SMF → Ca^2+^↓ (29)
		low-frequency AMF (21-kHz, 3.8mT)	No influence on T cells in rats (1 h/day, 14 days) (30)
		EMF (50 Hz, 100 μT, 60 days in rats) (with human serum albumin stimulation)	Splenic and thymic T-bet and GATA-3↑ Serum IFN-γ, IL-4 ↑ (31)
		Constant MF (60 μT) + alternating MF (100 nT)	TNF-α, IFN-γ ↑ in mice (20)
		MF + Iron-dextran nanoparticles	TCR clustering, T-cell activity, tumor killing ability ↑ (32)
	B-cells	Radiofrequency EMF (900 MHz, 2 h/day, 4 wks)	No differences on B-cells differentiation and antibody production in mice (33)

SMF, static magnetic field; AMF, alternating magnetic fields; EMF, electromagnetic field; MDSCs, Myeloid-derived Suppressor Cells; NK cells, natural killer cells.

**Figure 2:**
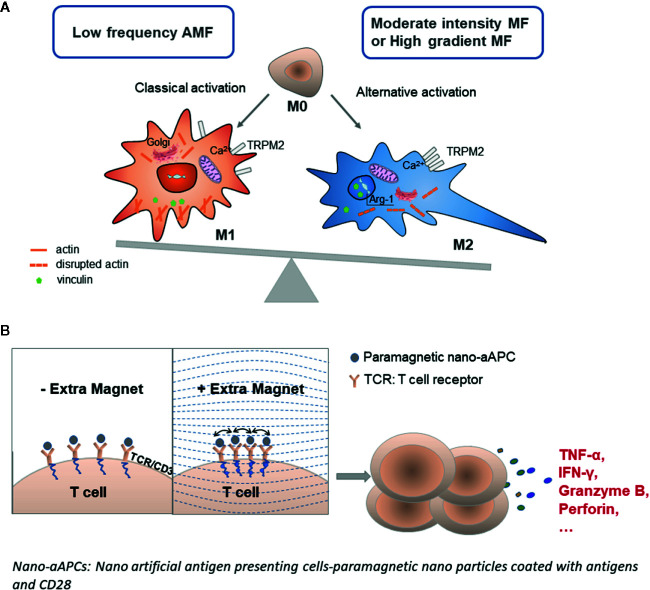
Molecular response of immune cells to the strength and frequency of the MF. **(A)** Macrophages are activated into the M1 phenotype by a low-frequency AMF and to the M2 phenotype upon exposure to a moderate-intensity MF or HGMF. The magnetic gradient force could cluster the cation channel receptor TRPM2 to disrupt the Ca^2+^ homeostasis; then the ion current-dependent actin polymerization is also affected, and the cell was reshaped subsequently. Vinculin, mitochondrial axis, and Golgi complex were also affected such that the Golgi complex in macrophages dispersed upon MF exposure. MF may also affect the nuclear actin in macrophage, that switched on the expression of some M2 macrophage-specific genes, such as Arg-1. **(B)** T-cell activation was largely increased under excess MF through accelerated TCR clustering when the cells were co-cultured with paramagnetic nano artificial antigen-presenting cells. Increased TCR clustering leads to enhanced downstream signaling, T-cell activation, and tumor killing capacity.

Macrophages originate from blood monocytes and further leave the circulation to differentiate in different tissues. They play key roles in normal tissue homeostasis, pathogen clearance, resolution of inflammation, and wound healing (35). Classification of macrophages can also be based upon polarization into M1 and M2 macrophages. M1 macrophages secrete proinflammatory cytokines, such as TNF-α and IL-1, while M2 macrophages secrete anti-inflammatory cytokines, such as IL-10 and TGF-β. M2 macrophages also produce either polyamines to induce proliferation or proline to induce collagen production, which further promotes wound healing and tissue repair. M1/M2 polarization is also involved in certain autoimmune diseases (36, 37). Based on several investigations so far, the polarization of naïve M0 macrophages into the anti-inflammatory M2 phase was largely influenced by moderate (around 0.6 T) or gradient (around 10^4^ Tm^-1^) MF (**Figure 2A**). The MF was reported to upregulate anti-inflammatory gene expression *via* activation of STAT6 and suppression of STAT1 in macrophages, thus facilitating the resolution of inflammation and wound healing (10) (**Table 1**). Therefore, an SMF has also been used to treat inflammatory diseases as an alternative therapy (10, 38, 39).

## Mechanical Deformation of Immune Cells With MFs

Notably, cells are weakly diamagnetic, but the culture medium can be more or less diamagnetic than the cells; the difference between susceptibilities of the cell and medium mainly determines the response of the cells to the MF (11). Apart from the susceptibility buoyance, the Lorentz force is also created when the MF interacts with the ionic currents in the membrane. The MF can be homogeneous and inhomogeneous (**Figures 1C, D**). Acting with these possible forces, an organism can experience unique effects under an SMF, namely HGMF, which could be a varying gradient MF created by using a Maxwell coil or a constant gradient MF that coexists with the homogeneous SMF at the off-center positions as shown in **Figure 1B**. An HGMF normally refers to a gradient higher than 10^3^ Tm^-1^ because, in such an MF, the cells would experience a magnetic gradient force with the same volume density as that of gravity (40). Apart from the macroscopic HGMF, magnetic NPs could also induce a local HGMF around single cells as reported by Tseng et al. Localized, NP-mediated magnetic forces were applied to HeLa cells through an MF with a gradient from 2.5×10^3^ Tm^−1^ to 7×10^4^ Tm^−1^ (41). When comparing with the homogeneous MF, an HGMF would have more pronounced biological effects on living cells, especially those of large size, such as macrophages. Wosik et al. find that magnet-exposed macrophages cluster the cation channel receptor TRPM2, which may render it nonfunctional and, thus, disrupt the Ca^2+^ homeostasis. This, in turn, affects the ion current–dependent actin polymerization and leads to macrophage elongation (11). These findings are very important to shed light on the various biological effects of MF observed in living cells. Within the cells, different cell components are subject to gradient forces of varying directions and strength; thus, the cells would reshape to adapt to the force and eventually end up balanced but distorted as shown in **Figures 1D** and **2A** (11, 23). The molecular responses involved in cytoskeleton remodeling, membrane potential change, and ion channel gating changes are addressed specifically in the next section.

In addition, when the HGMF strength and exposure is precisely controlled, these cellular effects could be used for immunoregulation such as regulating the opening/closing of ion channels (Figs. 1C, D; 2A). As an HGMF would have more pronounced biological effects on cells with large size, we speculate that an HGMF may also play a role in the differentiation of B-cells into plasma cells and the antibody production process. This might be interesting and worth following in a future study. Regarding the MFs effect on T-cells, as the cells loaded with magnetic NPs exhibit a “pull-in” instability under the largest gradient, Perica et al. have already used paramagnetic NPs to stimulate T-cells under an external MF, which resulted in clustered TCR size and downstream TCR signaling (**Figure 2B**). Using this approach, the authors have increased the T-cell expansion *in vitro* and antitumor activity, but they did not consider the NP-induced local HGMF in this case (32), which could be measured or calculated to build a quantitative stimulation model for future studies. Additionally, under an ultra-high SMF up to 27 T, a biased arrangement of chromosomes on the metaphase plate during mitosis was reported (42), which indicates that magneto-mechanical stress is able to assist in the division of cells under MF.

## Molecular Response of Immune Cells to the Strength and Frequency of MF

The high-strength MF (>10 T) could affect the charge (spin) transfer within radical pair reactions by changing the spin-state levels, thus influencing the rate of the biochemical reactions (43). On the other hand, AMF of low strength could also affect the reactions by altering the initial population of energy levels when the frequency is coherent. Such biochemical effects are translated into cellular responses at the molecular level. Therefore, the effect of MFs on immune cells is easier to observe in the cell activation processes under extra stimuli, which initiates a series of intracellular responses. The intracellular and intercellular free radicals and molecules, such as O_3_, NO, NO_2_, and FeCl_3_, are paramagnetic and can be redistributed by both the Lorentz force and the magnetic gradient force as known from the concepts of electrochemistry (44). During this process, cell components, such as the actin cytoskeleton, the Golgi complex, and the cation channel receptor TRPM2, are rearranged by the gradient force (11). In this case, when compared to resting lymphocytes, the lymphocytes under phytohemagglutinin stimulation were more sensitive to MF exposure, which accelerated their activation and exhaustion.

Take the macrophage as an example, Wosik et al. intensively investigated the macrophage polarization under MFs, and they observed the macrophage cluster the cation channel receptor TRPM2 to disrupt the Ca^2+^ homeostasis, then the ion current–dependent actin polymerization was also affected, the cell was reshaped subsequently (**Figure 2A**). Vinculin is an actin-dependent molecule that is important for cell focal adhesions, especially in the small GTPase RhoA-mediated pathway. Wosik et al, also find that extra MF exposure affects the vinculin distribution more in the cytoplasm and nuclei, and the RhoA-deletion macrophage accumulates the vinculin-rich focal adhesions more into the tail permanently; this causes an inability of the tail to detach from the substrate when the front of the macrophage is moving forward (45). In contrast, the aggregation of vinculin in the macrophage nuclei under MF exposure suggests that MF may change not only the cytoplasmic but also nuclear actin. The cell nucleus also contains a pool of nuclear actin that participates in regulation of chromatin status and gene transcription. In this way, MFs may influence the actin-binding molecules, such as vinculin, and cause its influx into the nucleus (11). MFs forced elongated macrophage switched on the expression of M2-specific genes, such as Arg-1, may also be related to the MF effect on nuclear actin. Interestingly, Yang et al. recently published very important results regarding the chirality-driven effect of static MFs on DNA synthesis. This newly discovered effect may provide fundamental knowledge for many MF-induced biological effects (46). An SMF of 27 T was reported to change the orientation and morphology of mitotic spindles in human cells, which suggests that the magnetic torque acts on both microtubules and chromosomes (42). In addition, the complete Golgi complex in the macrophage also dispersed upon MF exposure although the number of Golgi complex decreased dramatically in RhoA-deletion macrophage (**Figure 2A**). Therefore, the MF gradient effect on macrophage is very similar to the RhoA interference approach to change their phenotype, function, and migration (11, 12, 47). Last, due to the strong natural magnetic moments of Fe atoms and ions, the AMF and ultra-high MF would have a strong influence on the spin (charge) transfer between ions, which is yet to be fully investigated.

Magnetoreceptor (MagR) might be another explanation for the MF effect on living cells. Xie and Zhang published in 2015 that IscA1, which belongs to the iron-sulfur cluster protein (Isc) family, could form a complex with another protein Cry4 IscA1 and showed a paramount function in the iron-sulfur cluster assembly and cascade reaction system and was reported to possess a putative magneto-receptor capability (48, 49). However, these studies are still very controversial. Several scientists thought that the complex would be too weakly magnetic to sense Earth’s MF, and many groups could not reproduce the former results from Zhang et al. (50–52). For better understanding of the effect of MFs on biological molecules or tissues, more studies from different disciplines, such as physics, computer science, biology, chemistry, and other related fields, are still required.

## Immune Regulation by Iron Metabolism and the Effect of MFs on Iron Metabolism

**Figure 3:**
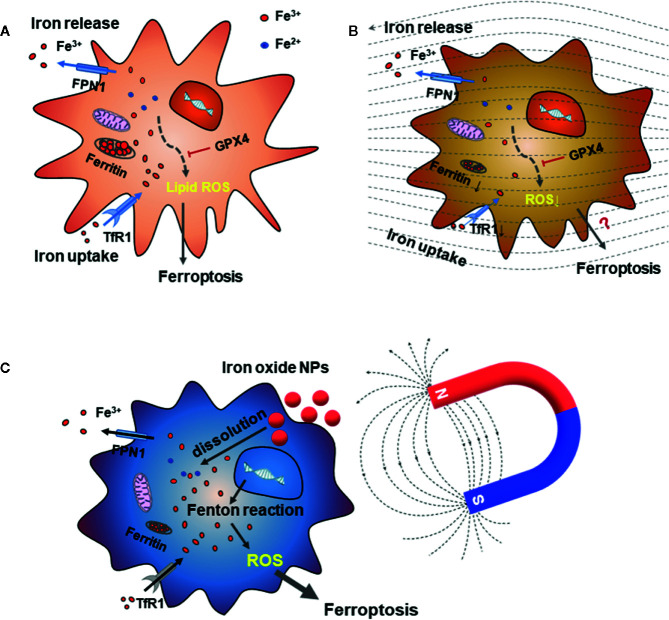
The effect of MFs on iron metabolism in macrophages and OCs. **(A)** Iron metabolism is tightly regulated in macrophages by the iron exporters, transferrin receptor 1 (TfR1, for iron uptake) and ferroportin-1 (FPN1, for iron release). In addition to iron utilization, the leftover cellular Fe^3+^ is stored in ferritin. Iron overload induced M1 polarization by increasing ROS production. On the other hand, accumulation of iron could cause the increased lipid ROS and ferroptosis, and GPX4 can inhibit the ferroptosis. **(B)** High SMFs can decrease the cellular iron content by reducing the expression of TfR1 and ferritin, thereby inhibiting the OC formation and resorption ability during the OC differentiation process. Mitochondrial concentration and ROS levels in OCs were also decreased under high SMF. **(C)** Strategies of the iron-based NPs for ferroptosis-based cancer therapy is shown in a cancer cell. Dissolution of iron oxide NPs can increase the cellular iron content and the following Fenton reaction-mediated ferroptosis-like cell death. Adapted from Wang et al. (54).

Iron, an essential nutrient that supports the immune system, exists in the form of heme in mammals and functions as a crucial redox catalyst in many cellular processes. Excess iron results in the formation of harmful hydroxyl radicals. Thus, iron metabolism, which extensively involves macrophages, is tightly regulated for immune homeostasis (53). More than 90% of the iron in an organism is supplied by the damaged or senescent red blood cells that are engulfed by macrophages, and most of the body iron is in the form of heme. Heme degradation can reduce inflammation, and the degraded products also show anti-inflammatory properties. The produced iron is either secreted from macrophages for the organism *via* the transmembrane protein ferroportin-1 (FPN1) or is stored intracellularly by ferritin (**Figure 3A**). The iron-storing capacity of ferritin is attributed to the ferroxidase activity of heavy chain H-ferritin (FTH), which converts reactive Fe^2+^ into Fe^3+^ so that iron can be stored in the ferritin mineral core and prevent the undesirable production of free radicals (55, 56). Heme catabolism is also found to contribute to macrophage polarization. Development of M1 macrophages is associated with intracellular iron retention by ferritin and reduced iron excretion *via* FPN1. Conversely, M2 macrophage polarization for tissue repair is associated with increased heme catabolism by heme oxygenase-1 (HO-1) and enhanced iron secretion *via* FPN1 (57, 58). MFs are reported to affect the expression of the iron-regulation proteins, such as transferrin receptor-1 (TfR1) and ferritin in osteoclasts (OCs), which are bone resorption cells derived from the monocyte/macrophage lineage (59–62). High SMFs of 16 T markedly inhibited iron absorption and iron storage-related protein expression, thus reducing the cellular iron content during OC differentiation and inhibiting the OCs formation (**Figure 3B**). On the other hand, a hypo-magnetic field exerted deleterious effects on osteoblast differentiation by simultaneously retarding alkaline phosphatase activity, mineralization, and calcium deposition with increased iron levels (60, 63). In addition, the mRNA expression of TfR1 was induced by a hypo-MF but was inhibited by a high MF. Therefore, iron metabolism in OCs can be regulated by altering the strength of the SMF. We speculate that, in macrophage, the effects of MF on iron metabolism should be similar. Recently, Zhou et al. (64) reported that iron overload induced M1 macrophage polarization by increasing ROS production and inducing p53 acetylation.

Nicotinamide adenine dinucleotide phosphate oxidase 2 (NOX2) is a transmembrane hemoprotein that transports electrons across the biological membrane and catalyzes the production of superoxide, •O2−. The accumulated •O2− can induce other ROS, including H_2_O_2_, which reacts with iron to generate hydroxyl radicals (•OH), hydroxide ions (OH−), and hydrogen peroxide radicals (HOO•) (65). An MF may also change the electronic spin state of radicals, ions, or triplet molecules, thus influencing the chemical activity of the corresponding compounds (66).

Furthermore, ferroptosis is a form of regulated cell death that is iron and ROS dependent and is distinguished from other forms of regulated cell death, such as apoptosis, autophagy, and necroptosis. Ferroptosis is initiated by oxidative perturbations of the intracellular microenvironment. Glutathione peroxidase (GPX4), heat shock protein beta-1, and nuclear factor erythroid 2-related factor 2 regulate ferroptosis negatively by limiting ROS production and reducing cellular iron uptake (67) (**Figure 3**). This process can be inhibited by iron chelators and lipophilic antioxidants (68). Several studies report that MFs are highly related with ferroptosis in tumor therapy. Yu et al. recently report that exogenous circularly polarized MF could enhance ferroptosis-like cell death–mediated immune response in cancer immune therapy (69). Yuan et al. also find that suppressing MF could determine cell fate through ROS-induced DNA damage, inducing oxidative stress and activation of the DNA damage repair pathways, eventually leading to apoptosis and ferroptosis (70). However, regarding the SMF’s effect on iron levels in OCs in **Figure 3B**, it is still not clear if there is some threshold about intensity of the SMFs over which the cellular iron could be increased high enough to induce ferroptosis of macrophages or OCs. Considering activation of mitochondrial voltage-dependent anion channels and mitogen-activated protein kinases, upregulation of endoplasmic reticulum stress, and inhibition of cystine/glutamate antiporter are also thought to be involved in ferroptosis, we speculate that MF induced-ferroptosis may be also related with both ion channels and iron metabolism. Cichon et al. report that low-frequency EMF could increase the expression of GPX4 and enhance the antioxidant defense of the body in poststroke patients, but they did not check the ferroptosis level of the cells (71, 72). Last but not least, MF-boosted ferroptosis-like cell death is an important new strategy for cancer therapy. This is mainly based on superparamagnetic iron oxide nanoparticles that induce ferroptosis *via* inorganic nanozyme-mediated catalysis and magnetotherapy by hyperthermia treatment (**Figure 3C**) (54, 69, 70, 73).

## MF-Related Immune Therapy

Based on most of the published literature so far, low-intensity MFs (<10 mT) slightly activate the immune response, and moderate-intensity MFs and HGMFs promote the immune response more toward anti-inflammatory directions though regulation of macrophage differentiation and other immune cells. The low-intensity MFs mainly promote the inflammatory responses, and the strong ones mainly promote the anti-inflammatory responses. Therefore, MF-related therapy has been applied as an adjuvant therapeutic option for inflammatory diseases (74). Shang and Song et al. develop a contractibility band with SMFs to improve skin wound healing (10) (75). Some researchers have also tried magnetic-assisted treatment for liver fibrosis and rheumatoid arthritis (14, 36, 38, 76). Magnetic seizure therapy (MST) is the use of transcranial magnetic stimulation to induce seizures for the treatment of neurological disorders (77–82). Moreover, magnetic-assisted treatment for inflammatory diseases varies depending on the MF dosing parameters. Thus, more precise, and large-scale investigations based on different types of MFs both *in vitro* and *in vivo* are still needed before any clinical translation process.

Importantly, the magnetic targeting technique for cell therapy is a new strategy to aid cell product delivery and increase the retention of the cells at the target, thus enhancing the therapeutic effects. Immunotherapy has gained increasing popularity in this decade due to its ability to regulate the immune system to fight against infections, cancers, or rejections in organ transplantations. Adoptive cell therapy is one of the most promising immunotherapies that transfer the ex vivo expanded or engineered cells into the patients to improve and enhance immune functionality. However, *in vivo* experiments show that only a small number of the transferred cells reach the target site, thus limiting the therapeutic effects of the cell therapy (83). When the therapeutic cells are magnetized with magnetic NPs or microparticles, an external MF could guide the cells into the target site *in vivo*. Therefore, using this method, the transferred cells can be directed and retained at the target site for effective and enhanced therapeutic effect. Silva et al. (84) report the use of this technique to improve the therapeutic effect of mesenchymal stromal cells. Jang et al. (85) also used this method to guide NK cells into the target tumor site, which enhanced the tumor killing capacity of NK cells by 17-fold. Similar approaches have also been used for T-cell therapy and DCs for more effective antigen delivery and immune stimulation (24, 39, 86). However, much work still needs to be conducted for its clinical application, for instance, the correct placement of the external magnets or the accurate and precise guiding of the cells in blood or through tissues (87, 88). In addition to the external guiding of cells, another important prospect for MF-related T-cell therapy is the paramagnetic NPs coated with antigens and CD28 that can cause TCR clustering and increase the T-cell activity for efficient tumor killing (32). Recently, as an important new strategy for cancer therapy, MF-boosted ferroptosis-like cell death in cancer cells has attracted increasing attention over the years. However, for the iron-based NPs to induce ferroptosis, the content of iron must be very high (75 mg/kg body weight) (89). To achieve this, additional components besides iron are needed to decrease the iron dose and make the ferroptosis cancer therapy more practical.

## Summary

The study of the effects of MFs on living cells has been an important research subject for decades. In this review, we mainly focus on the effects of MFs on the innate immune system. To date, the most investigated immune cells in MFs are the innate immune cells, namely monocytes and macrophages. Here, we summarize the effect of MFs on macrophage differentiation and iron metabolism upon exposure to moderate/strong SMFs and HGMFs. Subject to the mechanical forces of varying directions and strength in MFs, the cells reshape to adapt to the force together with cytoskeleton remodeling, membrane potential change, and ion channel gating changes. These alterations are very similar to changes caused by the small GTPase RhoA interference in macrophages. In addition to the mechanical forces in MFs, iron metabolism may also contribute to the M2 macrophage polarization because similar effects are observed in OCs, which derive from monocytes/macrophage lineage (57, 58, 60, 63). Therefore, we speculate that MFs may also play a role in affecting the iron metabolism, including ferroptosis in macrophage differentiation, which is worthy of investigation in the future. Until now, studies on the effect of MFs on adaptive immune cells were mainly performed on T-cells coated with paramagnetic NPs, which is very promising for cell therapy as external magnets might not only guide the transferred T-cells into the target site *in vivo*, but also cluster the TCR of cells, thus increasing the cell–cell contact and communication for subsequent activation and function (32). Moreover, the safety of the materials should be evaluated before use as advanced MNPs are yet to be developed. On the other hand, as large cells are more sensitive to HGMFs, we speculate that process such as B-cell maturation into plasma cells and antibody production might also be affected by HGMFs.

In summary, to understand the effect of MFs on immune cells, both experimental research and theoretical modeling are essential. High-tech instruments are also indispensable for such experimental research. Evidence for cytoskeleton remodeling was provided by direct confocal images of F-actin organization in cells exposed to MFs (90, 91). A combination of scanning tunneling microscopy with 0.4 T SMF can even show the structure, morphology, and dynamics of a protein molecule in solution (92). Liquid-phase scanning tunneling microscopy can provide direct evidence for the effect of MFs on proteins under physiological conditions. Additionally, theoretical modeling from other related disciplines to mimic the cellular interaction and signaling in MFs should be performed together with experimental research to elucidate the intracellular and intercellular immune responses. Therefore, it is essential for multidisciplinary researchers to collaborate for better understanding of the MFs effect on immune regulation.

## Author Contributions

All authors listed have made a substantial, direct, and intellectual contribution to the work and approved it for publication.

## Funding

This work was supported by Natural Science Basic Research Program of Shaanxi (Program No. 2020JQ-520), National Key R&D Project of China (Grant No. 2018YFC0115300), the Strategic Priority Research Program of the Chinese Academy of Sciences (Grant No. XDB30000000) and the Innovation Capacity Support Plan of Shaanxi Province (No. 2020TD-040).

## Conflict of Interest

The authors declare that the research was conducted in the absence of any commercial or financial relationships that could be construed as a potential conflict of interest.

## Abbreviations

AMF, alternating magnetic fields; DC, dendritic cell; EMF, electromagnetic field; FTH, heavy chain H-ferritin; FPN1, ferroportin-1; GPX4, glutathione peroxidase; HGMF, high-gradient magnetic field; HO-1, heme oxygenase-1; MDSC, myeloid-derived suppressor cell; MF, magnetic field; MRI, magnetic resonance imaging; NK cell, natural killer cell; NP, nanoparticle; OC, osteoclast; ROS, reactive oxygen species; SMF, static magnetic field; TCR, T-cell receptor; TfR1, transferrin receptor-1.
